# Transparency in Admissions and Personalized Learning Through Resident Patient Selection

**DOI:** 10.31486/toj.21.0066

**Published:** 2022

**Authors:** Andrea Archibald, Paul Zimmerman, Winn Seay, Lalit Verma, Jonathan Wilson, Poonam Sharma

**Affiliations:** ^1^ Department of Medicine, Duke Regional Hospital, Durham, NC; ^2^ Department of Medicine, Duke University Hospital, Durham, NC; ^3^ Department of Biostatistics and Bioinformatics, Duke University, Durham, NC

**Keywords:** *Education*, *internship and residency*, *patient selection*, *professional autonomy*, *triage*

## Abstract

**Background:** Adult learning (andragogy) posits that adult learners have an improved educational experience when engaged in self-directed learning. The decision to allocate patients to the teaching service vs a nonresident service varies according to institution. Previously, our institution focused on faculty perception of learning value as the deciding factor in patient assignment. We hypothesized that transitioning to a process in which adult learners (residents) select patients for their teams based on their own identified learning needs could improve the educational experience without adversely impacting the workflow for nonteaching teams.

**Methods:** A new patient assignment model focused on learner-driven identification of patients for their own inpatient service, consistent with the principle of andragogy, was created. This patient assignment strategy was tested during a 1-month pilot period followed by a 5-month implementation period with 20 senior residents and 31 hospitalists. Both residents and hospitalists were surveyed after the intervention.

**Results:** Sixteen of 20 residents completed the paper survey, and 100% of the respondents indicated “yes” when asked if they were able to direct cases to their team that were in line with their learning goals and if the new process should continue. Twenty-one of 31 hospitalists responded to the electronic survey; 81% of responding hospitalists reported a slightly positive to very positive impact on the hospitalist workflow, and 76% felt the new process should continue. The new patient assignment model had no negative impact on case mix index or length of stay.

**Conclusion:** Restructuring patient assignment processes based on educational theory may improve resident education and improve hospitalist workflow.

## INTRODUCTION

One tenet of adult learning theory (also called andragogy) posits that adult learners have an improved educational experience through self-directed learning with internal, rather than external, motivators.^1^ While medical education has both internal and external motivators, there is opportunity to improve and encourage more autonomy and self-guided learning. One example is patient assignment to resident teaching teams. To meet the volume of inpatient care needs with a limited number of residents, many hospital systems have developed both resident and nonresident services, with nonresident services providing care directly via an attending or an advanced practice provider.^2^ Patient allocation to the resident teaching service or nonresident service varies according to institution. Despite large institutional variation, acuity, time of admission, and locations of available beds are factors that can impact assignment.^2^ Our institution used a time-based assignment process with the hospitalist's perception of learning value as a deciding factor in patient assignment, a process that left limited opportunity for the adult learners (the residents) to give voice to their own learning goals prior to patient assignment.^3^ Such systems may miss an opportunity for resident engagement to drive their own learning and can result in a negative perception of patient distribution. The limitations of a faculty-led assignment process have been described and include resident perceptions of inequity, even when such inequity does not exist.^4^

## METHODS

This educational study was conducted at a 369-bed tertiary-care community hospital associated with an academic medical center. The project received an exemption from the institutional review board. In the patient assignment model prior to intervention, a triaging hospitalist received the pages for admission and then assigned patients to the resident teaching team at his/her discretion with the loose criteria of *good learning value* and assignment within the timeframe for admissions. A new patient assignment strategy was created with the goals of facilitating resident engagement and encouraging self-directed learning. In the new patient assignment process, the resident of the on-call team received the identical page as the triaging hospitalist about each new admission from the emergency department. The resident could then briefly review the patient chart, discuss the patient with the students on the team, and consider the learning goals and objectives of the team. The resident notified the hospitalist if the team would admit a particular patient or if they would prefer the nonresident service to assume care. Resident teams had clearly designated requirements for total number of admissions each call day that did not change before or after the intervention. In addition, residents had clearly defined windows of time for accepting admissions that also did not change. Of note, because of the complexities of patient assignment, hospital capacity, and workflow, the hospitalist retained final say in the disposition of patients to teaching vs nonteaching teams.

Hospitalists were educated about the new workflow via monthly group meetings and educational emails, and residents were educated about the process during their rotation orientation. The new patient assignment strategy was tested during a 1-month pilot (July 1 to July 31, 2018), followed by a 5-month implementation period (August 1 to December 31, 2018).

**Figure 1.**
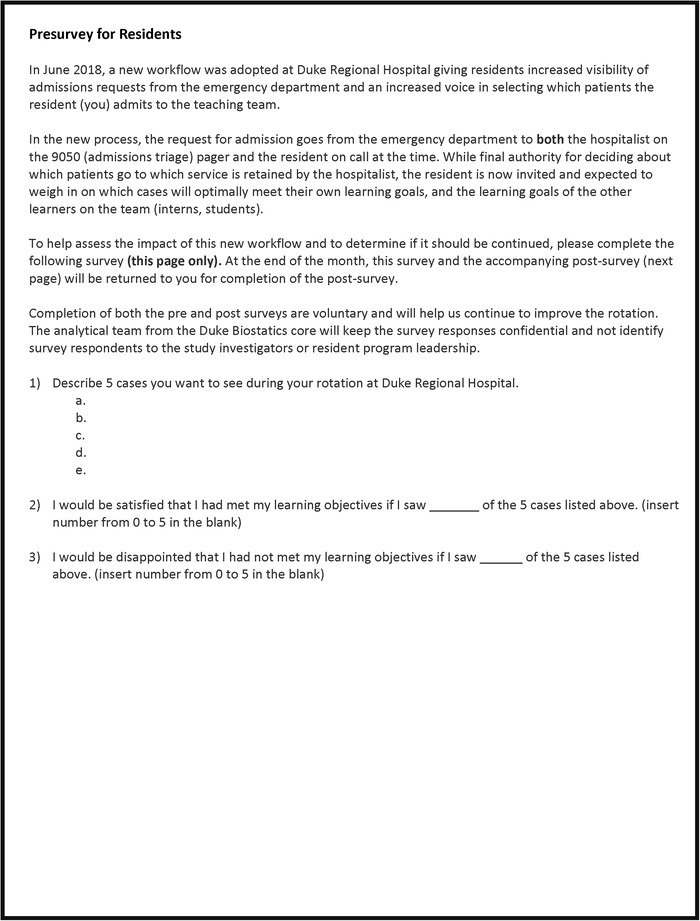
Resident presurvey.

**Figure 2.**
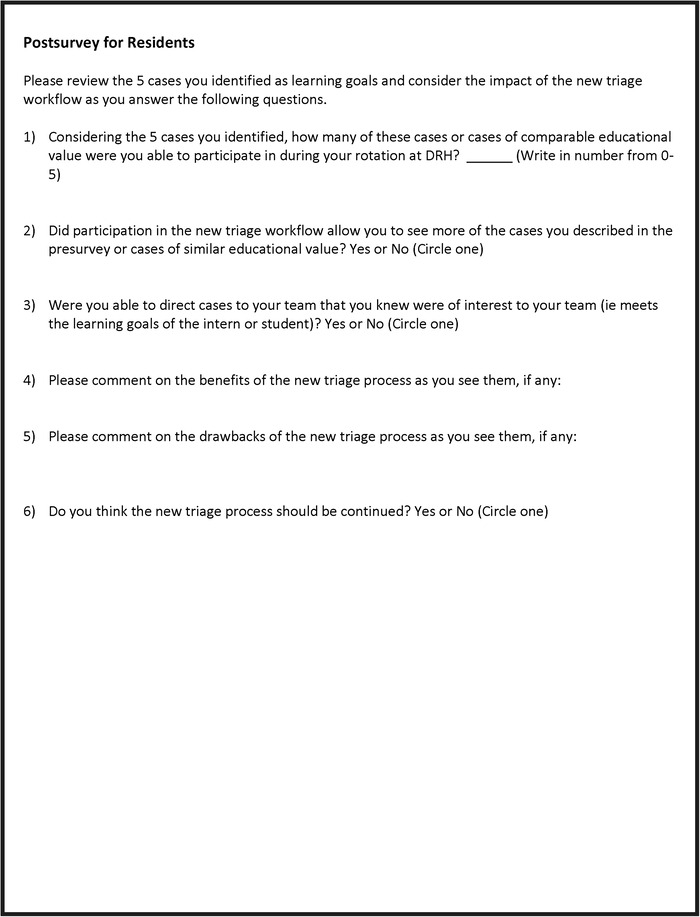
**Resident postsurvey.** DRH, Duke Regional Hospital.

To assess the impact of the intervention on the educational experience, at the start of each block, residents were asked to identify on a paper presurvey 5 cases they would like to see during the rotation (Figure 1). A paper postsurvey at the end of the block asked how many of the cases they had previously identified (or cases of comparable educational value) they were able to participate in during their rotation (Figure 2). Residents were also asked in a yes/no format if participation in the new triage workflow process allowed them to see more of the cases they identified in the presurvey (or cases of comparable educational value), if they were able to direct cases to their team that were of interest to them, and if they thought the new process should be continued. Two additional questions invited write-in comments on the benefits and drawbacks of the new system.

**Figure 3.**
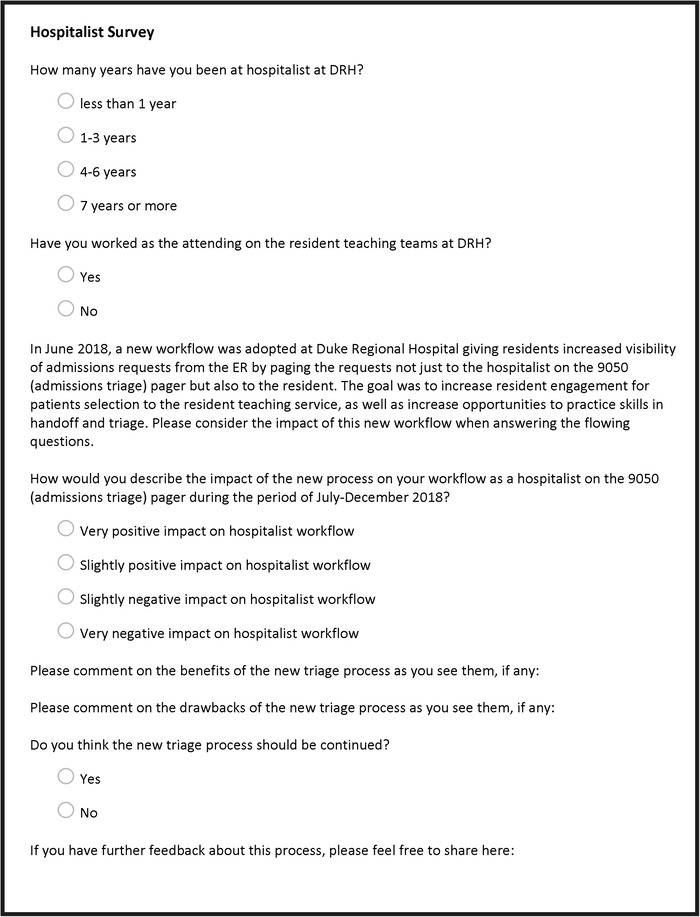
**Hospitalist survey.** DRH, Duke Regional Hospital; ER, emergency room.

Impact of the intervention on hospitalist workflow was assessed using an electronic survey of hospitalists at the end of the implementation period (Figure 3). The surveys were developed with a statistician to ensure interpretability.

The study participants were 20 internal medicine residents in their final year of residency, rotating in 1-month blocks on an inpatient internal medicine rotation. This rotation generally does not include second-year residents. Each resident team also included an intern and students. Because of scheduling logistics, students and interns were not included in the survey. The 31 hospitalists who interacted with the resident teams during the study period either as the attending on the teaching team or as the admitting triage hospitalist were invited to complete the postintervention survey.

We also reviewed the case mix index (CMI) and length of stay (LOS) for the teaching and nonteaching services. Cases were categorized by teaching status (teaching or not) and time period: August 1 to December 31, 2017 (1 year prior to study period) and August 1 to December 31, 2018 (study period). To test for consistency across time periods, a noninferiority test was used with a margin of ±1 day for the LOS outcome. Equivalency of CMI across time periods was examined using the two-one-sided tests (TOST) approach, with an equivalency margin of 0.15 CMI units. Using the TOST approach, two-one-sided 95% confidence intervals were used to construct an equivalency interval that could then be compared against the equivalency margins, in this case –0.15 to 0.15. Of note, the margin for CMI and LOS is negative because of the difference being taken as prior year period minus resident triage study period; negative values indicate longer LOS in the period that resident triage was implemented. We also compiled the 10 most frequent discharge diagnoses in both time periods.

## RESULTS

Of the 20 residents who participated in the educational triage system during the 5-month implementation period, 17 completed at least part of the follow-up survey and 16 completed the entire survey, for a response rate of 80%. Results from the yes/no resident survey questions are presented in Table 1, and representative comments from the postsurvey are presented in Table 2. One hundred percent of the responding residents indicated “yes” when asked if they were able to direct cases to their team that were in line with their learning goals and if the new process should continue. The resident comments also demonstrated a positive response and included themes of diversity of cases, control of workflow, improved autonomy, and facilitation of the education of other learners (interns, students).

**Table 1.** Postsurvey Resident Responses to Yes/No Questions

Survey Question	Total Responses	Proportion of Yes Responses	95% CI^a^
Did participation in the new triage workflow allow you to see more of the cases you described in the presurvey or cases of similar educational value?	16	0.9375	0.6977, 0.9984
Were you able to direct cases to your team that you knew were of interest to your team (ie, meets the learning goals of the intern or student)?	17	1.000	0.8049, 1.000
Do you think the new triage process should be continued?	17	1.000	0.8049, 1.000

^a^Because of the small sample size, the 95% CIs for the proportions were created using exact binomial limits (Clopper-Pearson).

**Table 2.** Postsurvey Representative Resident Comments

“Increased resident autonomy. Allows better timing of resident team admissions to optimize education.”
“Improves camaraderie and culture between residents and hospitalists.”
“I love it! It helps to manage workflow of your team which adds to the organizational skills as a [senior resident]. It also allowed me to ask my intern, “Have you seen x, y, z before?” and take patients of interest to them.”
“Cases that were great learning cases and ones that provided great discussion were shunted over to the teams. This made rounds more interesting and fun. It also allowed for variety and diversity of cases.”

Of the 31 hospitalists who participated in the educational triage system during the 5-month implementation period, 21 completed the postintervention survey, for a response rate of 68%. Seventeen of the 21 hospitalists (81%) reported a slightly positive to very positive impact on the hospitalist workflow, and 16 of 21 hospitalists (76%) felt the new process should continue.

The results for CMI and LOS are shown in Table 3. Two cases had negative LOS and were removed from the analysis of that variable. One outlier case (LOS >1 year) was included in the primary analysis, but a sensitivity check with it removed was done as well. This outlier appeared in the prior year teaching cases.

**Table 3.** Hospital Metrics in Prior Year Compared to Resident Triage Study Period

		1-Year Prior Period^a^		Resident Triage Study Period^b^		
Group	Measure	n	Mean (SD)	n	Mean (SD)	Estimate (Equivalency Interval)^c^
Teaching group	Length of stay	672	6.03 (17.04)	660	4.88 (6.90)	1.15 (–0.02, infinity)
	Length of stay, outlier removed^d^	671	5.44 (7.36)	660	4.88 (6.90)	0.56 (–0.09, infinity)
	Case mix index	673	1.39 (0.80)	660	1.39 (0.77)	0 (–0.07, 0.07)
Nonteaching group	Length of stay	1,998	5.38 (6.64)	2,590	4.90 (4.79)	0.48 (0.20, infinity)
	Case mix index	1,999	1.45 (0.95)	2,590	1.42 (0.93)	0.03 (–0.01, 0.08)

^a^The 1-year prior period is August 1 to December 31, 2017.

^b^The resident triage study period is August 1 to December 31, 2018.

^c^Difference of prior year period minus resident triage study period.

^d^Length of stay >1 year.

Note: n=number of patients.

Among patients seen by the teaching service, LOS was no more than 1 day longer in the resident triage study period vs the prior year period. Table 3 shows the estimate of 1.15 days and a lower 95% one-sided interval value of –0.02. This result is consistent when the outlier case is removed and is also consistent with the estimate for the nonteaching patients during the same time periods.

The TOST equivalency interval for CMI (–0.07, 0.07) is fully contained within the stated equivalency margins of –0.15 to 0.15, showing evidence of the equivalency of CMI values across the time points in the teaching group. The result is consistent across the teaching and nonteaching group cases.

We tracked the top 10 discharge diagnoses for the teaching service in the year prior (August 1 to December 31, 2017) and during the study period (August 1 to December 31, 2018). The results are listed in Table 4 and show that 8 of the top 10 diagnoses did not change. The top 10 discharge diagnoses were representative of the most common diseases treated by inpatient general medicine services.

**Table 4.** Top 10 Discharge Diagnoses on the Teaching Service by Time Period

1-Year Prior Period^a^	Resident Triage Study Period^b^
1. Sepsis, unspecified organism	1. Sepsis, unspecified organism
2. Hypertensive heart and chronic kidney disease with heart failure and stage 1 through 4 chronic kidney disease, or unspecified chronic kidney disease	2. Hypertensive heart and chronic kidney disease with heart failure and stage 1 through 4 chronic kidney disease, or unspecified chronic kidney disease
3. Chronic obstructive pulmonary disease with (acute) exacerbation	3. Chronic obstructive pulmonary disease with (acute) exacerbation
4. Hypertensive heart disease with heart failure	4. Acute kidney failure, unspecified
5. Acute kidney failure, unspecified	5. Hypertensive heart disease with heart failure
6. Urinary tract infection, site not specified	6. Pneumonia, unspecified organism
7. Non-ST elevation myocardial infarction	7. Other acute pulmonary embolism without acute cor pulmonale
8. Pneumonia, unspecified organism	8. Type 1 diabetes with ketoacidosis without coma
9. Pneumonitis due to inhalation of food and vomit	9. Non-ST elevation myocardial infarction
10. Acute and chronic respiratory failure with hypoxia	10. Hypertensive heart and chronic kidney disease with heart failure and with stage 5 chronic kidney disease, or end stage renal disease

^a^The 1-year prior period is August 1 to December 31, 2017.

^b^The resident triage study period is August 1 to December 31, 2018.

## DISCUSSION

A patient assignment model built to focus on the strengths of adult learning (andragogy) shifted patient selection from faculty to residents and improved resident autonomy, allowed residents to select high-yield medical diagnoses for the team, and improved overall resident experience. Based on our survey responses, this improvement did not come at the cost of hospitalist experience or hospital metrics. In fact, the delegation of the responsibility from hospitalist to resident was well-received by both hospitalists and residents. While our study did not investigate why hospitalists thought the new workflow should be continued, possible explanations include (1) observation of benefit to resident teams either during the triage role or during their time as an attending on the resident team, and (2) decreased cognitive load for the triaging hospitalist.

Interestingly, the most common discharge diagnoses on the teaching service remained largely the same before and during the intervention period. This finding aligns with prior research showing that while a faculty-led decision model may lead to resident misperception, residents and faculty agree on many characteristics of a good teaching case.^4^ In addition, 8 of the top 10 discharge diagnoses (sepsis, heart failure with or without renal failure, pneumonia, chronic obstructive pulmonary disease, renal failure, and diabetes complications) during the resident triage study period were among the most common diseases treated by inpatient general medical services, indicating resident patient selection did not change the breadth of exposure necessary for a thorough general medicine experience.^5^

One limitation of this study is that the intervention was implemented at a single site. Replication at other locations would strengthen the results. Different sites may have differences in culture, admission number expectations, and communications that may require adaptations. Another limitation is the response rate of the hospitalists. Given the small total number of hospitalists, even a few more responses could have had a considerable impact. However, we think this study is a good proof of concept that educational theory can be a platform for designing workflow structures to optimize educational experience without negatively impacting—and possibly even improving—hospitalist workflow and metrics. The new patient assignment strategy has continued since the intervention period during a time of change and expansion of the teaching services, strengthening the idea that this system is robust and sustainable.

One area of future study is to better assess the true learning value of resident triage by conducting an in-depth review of the types of patients and diagnoses the residents choose. Another possible area of study is the extent to which this process gives residents increased visibility into all admissions to the hospital medicine service. The literature about the role of the triage hospitalist and the need to explicitly develop resident skills related to assessing patients for inpatient admission is increasing.^6,7^ This patient allocation strategy could be a platform for working with residents to develop these skills.

## CONCLUSION

Incorporating the perspective of andragogy as a guiding principle offers an opportunity to align resident educational needs and hospitalist workflows. A redesigned patient assignment strategy consistent with andragogy was well received by residents and hospitalists and did not negatively impact other measures of patient care.

## Notes

*This article meets the Accreditation Council for Graduate Medical Education and the American Board of Medical Specialties Maintenance of Certification competencies for Patient Care, Medical Knowledge, and Practice-Based Learning and Improvement.*
